# Lower Circulating Lymphocyte Count Predicts ApoE **ε**4‐Related Cognitive Decline in Parkinson's Disease

**DOI:** 10.1002/mds.28799

**Published:** 2021-10-13

**Authors:** Kazuto Tsukita, Haruhi Sakamaki‐Tsukita, Ryosuke Takahashi

**Affiliations:** ^1^ Department of Neurology, Graduate School of Medicine Kyoto University Kyoto Japan; ^2^ Advanced Comprehensive Research Organization Teikyo University Tokyo Japan; ^3^ Division of Sleep Medicine Kansai Electric Power Medical Research Institute Osaka Japan

Neuroinflammatory changes in the brain, including infiltration of lymphocytes, particularly T cells, play a critical role in the pathogenesis of Parkinson's disease (PD).^1^, ^2^ Interestingly, in the peripheral blood of PD patients, a decrease in circulating lymphocyte counts occurs, mainly due to a decrease in T cells.^1^, ^3^ Furthermore, it has recently been reported that lower lymphocyte count might be causally related to the subsequent development of PD.^4^ Inspired by these observations, we aimed at assessing whether low lymphocyte count is associated with the subsequent development of the key milestones in PD's disease course, specifically cognitive impairment, with a particular attention to the apolipoprotein E (ApoE) ε4 allele, a crucial modifying factor in cognitive impairment.^5^, ^6^

In this retrospective cohort study, using the Parkinson's Progression Markers Initiative data, 167 de novo PD patients were enrolled (Fig. S1) and were followed up for 2 years (Tables S1 and S2; Text S1). R scripts made for the analysis are freely available at http://dx.doi.org/10.17632/7s8sng9yn8.2 or https://github.com/KazutoTsukita/Mov_Disord_2021.

We primarily used the multivariate linear mixed‐effects model adjusted for various covariates (age, sex, levodopa‐equivalent dose, disease duration, and baseline severity of smell deficit and rapid‐eye‐movement sleep behavior). We observed that only in PD patients carrying ApoE ε4 allele, baseline lymphocyte count had significant interaction effect on the longitudinal decline in the Montreal Cognitive Assessment (MoCA) total score, such that lower baseline lymphocyte count was associated with accelerated MoCA score decline (carrier, the standardized fixed‐effects coefficient of the interaction term (β_interaction_) = 0.17 [95% confidence interval, CI: 0.04, 0.30], *P* = 0.01; noncarrier, β_interaction_ = −0.00 [95% CI: −0.10, 0.09], *P* = 0.94). When PD patients, with and without ApoE ε4 allele, were dichotomized using the median of baseline lymphocyte count (carrier, 1.72 × 10^3^/μL; noncarrier, 1.74 × 10^3^/μL) (Table S3), the interaction effect was apparent only in PD patients carrying ApoE ε4 allele (carrier, β_interaction_ = 0.45 [95% CI: 0.20, 0.71], *P* < 0.001; noncarrier, β_interaction_ = −0.03 [95% CI: −0.22, 0.15], *P* = 0.72) (Fig. 1A,B). The interaction effects of baseline lymphocyte count on the progression of specific domains of cognitive impairment did not reach statistical significance (Fig. 1C). Sensitivity analyses confirmed the robustness of our result in a range of follow‐up periods (Table S4) and even when missing values were imputed (Table S5).

**FIG. 1:**
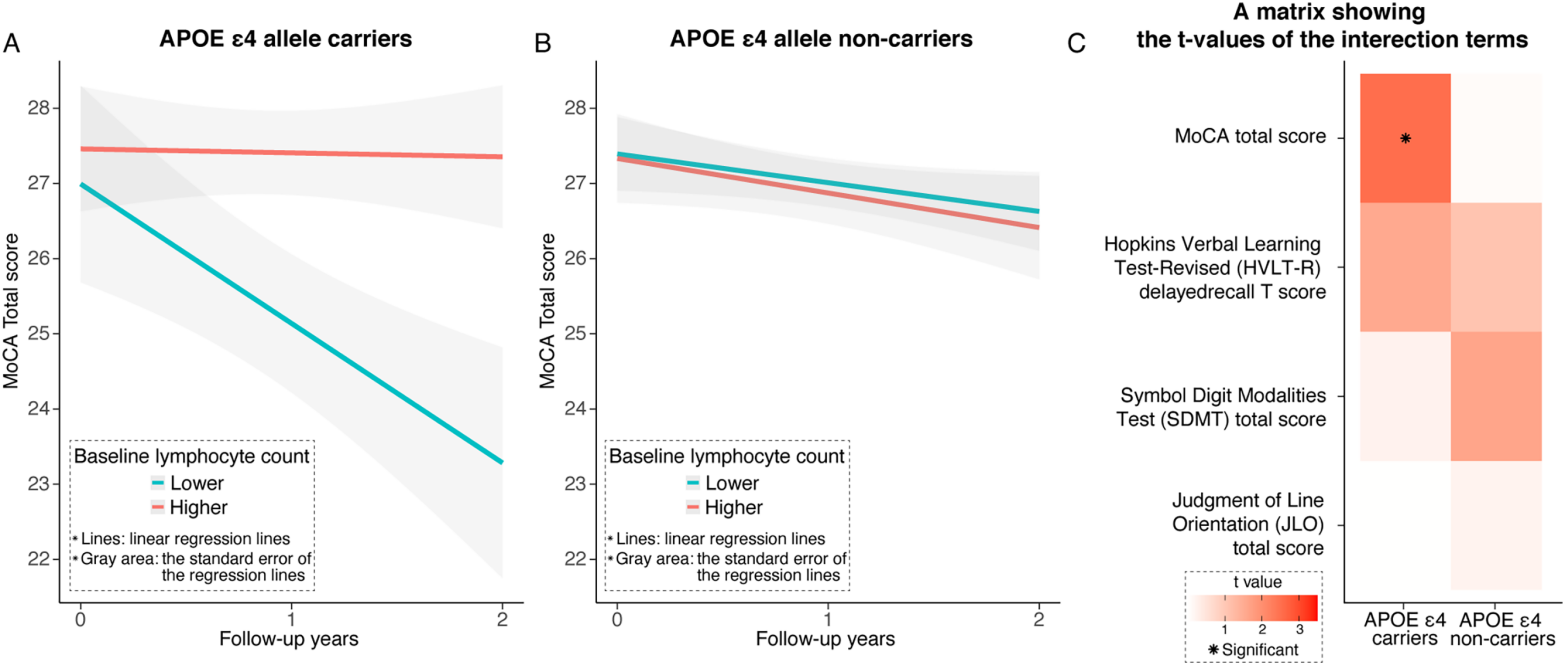
Evaluations of interaction effects of baseline lymphocyte counts on cognitive decline in patients with PD (**A**) with or (**B**) without the ApoE (apolipoprotein E) ε4 allele and (**C**) those on the progression of specific domains of cognitive impairment. [Color figure can be viewed at wileyonlinelibrary.com]

An interesting aspect of the present result is that baseline lymphocyte count was clearly associated with subsequent cognitive decline only in PD patients carrying ApoE ε4 allele. Given the importance of ApoE ε4 allele in blood–brain barrier (BBB) dysfunction and the role of circulating T cells in PD pathogenesis (Text S2),^1^, ^7^ our result might indicate the cooperative pathological role of BBB dysfunction and circulating lymphocytes in PD. Alternatively, the brain cortex of patients carrying ApoE ε4 allele may be particularly vulnerable to lymphocyte infiltration. Admittedly, this study has some limitations (Text S3); however, because many covariates were adjusted for, we believe that our result indicates that biological phenomenon reflected by the decrease in the lymphocyte count might actively exacerbate the pathology driving cognitive dysfunction in synergy with the APOE ε4 allele, thereby providing important clinical and pathophysiological implications.

## Author Roles

(1) Research project: A. Conception, B. Organization, C. Execution; (2) Statistical analysis: A. Design, B. Execution, C. Review and critique; (3) Manuscript preparation: A. Writing of the first draft, B. Review and critique.

K.T.: 1A, 1B, 1C, 2A, 2B, 2C, 3A, 3B

H.S.‐T.: 1A, 1B, 1C, 2A, 2B, 2C, 3A, 3B

R.T.: 1A, 3B

## Full financial disclosures for the previous 12 months

This work was supported by JST [Moonshot R&D][Grant Number JPMJMS2024].

K.T. and H..S.‐T.: nothing to report. R.T.: research grants and consultation fees from Takeda Pharma, Boeringer Ingelheim, Dainippon Sumito Pharma, Kyowa‐Kirin Pharma, Eisai Pharma, Otsuka Pharma, Novartis, Sanofi, Kan Institute, and Nihon Medi‐physics; research grants from Astellas Pharma; and consultation fees from AbbVie, Mylan, JBO, Sanwa Kagaku, FP Pharma, Tsumura, Kissei, Chugai Pharma, and Biogen, outside the submitted work. The remaining authors (K.T. and H.S.‐T.) have no conflicts of interest to declare.

## Supporting information


**APPENDIX S1**. Supporting Information.

## Data Availability

Data used in this retrospective cohort study were obtained from the Parkinson's Progression Markers Initiative (PPMI) database (www.ppmi-info.org/data) on July 28, 2021. For up‐to‐date information on the study, visit www.ppmi-info.org. R scripts made for the analysis are freely available at http://dx.doi.org/10.17632/7s8sng9yn8.2. or https://github.com/KazutoTsukita/Mov_Disord_2021.
