# Supplementary material for: Lower Circulating Lymphocyte Count Predicts ApoE ε4‐Related Cognitive Decline in Parkinson's Disease
Source: Mov Disord. 2021 Oct 13;36(12):2969–71. doi: 10.1002/mds.28799 (PMC9293429; doi:10.1002/mds.28799)
Supplement: Supplementary file 1 — APPENDIX S1. Supporting Information. [file MDS-36-2969-s001.docx]

**SUPPLEMENTARY TEXT**

**Text S1.** Supplementary methods.

**Study participants and clinical evaluation**

Data used in this retrospective cohort study were obtained from the Parkinson’s Progression Markers Initiative (PPMI) database ([www.ppmi-info.org/data](http://www.ppmi-info.org/data)) on July 28, 2021. For up-to-date information on the study, visit [www.ppmi-info.org](http://www.ppmi-info.org/). Participants in the original PPMI study included de novo patients with PD who were not on dopaminergic medication and exhibited presynaptic dopaminergic terminal loss as confirmed by dopamine transporter imaging (<http://www.ppmi-info.org/study-design>).

Among the patients whose data were registered in the PPMI database, we enrolled patients with de novo, drug-naïve PD based on the availability of information pertaining to the APOE genotype, baseline Montreal Cognitive Assessment (MoCA) score, and baseline lymphocyte count.

Since loss to follow-up of >20% poses serious threats to study validity,^1^ we primarily used 2-years as the follow-up period in this study (Table S1)

**Ethical statements**

Each Parkinson’s Progression Markers Initiative (PPMI) participating site received approval from their local ethic committee prior to study initiation, and written informed consent was obtained from all subjects prior to participation. Our study adheres to the publication policy in the PPMI study (<https://www.ppmi-info.org/documents/ppmi-publication-policy.pdf>) and we have obtained permission for publishing our research by the Data & Publication Committee of the PPMI study.

**Clinical evaluations**

In addition to age, sex, disease duration (time since the onset of diagnosis), and Hoen-Yahr stage, we extracted the baseline and annual follow-up data pertaining to motor and cognitive function, autonomic symptoms, sleep-related symptoms, and levodopa equivalent daily dose.

We assessed the global motor function in the “off” state using the MDS-UPDRS part III score. In the PPMI study, the “off” state was defined as the state that occurred after the patients had withheld their dopaminergic medication for at least 12 hours.

We also assessed global cognitive function using the MoCA. To assess the sub-domains of cognitive function, we employed the delayed recall T score of the Hopkins Verbal Learning Test-Revised as a measure of verbal recent memory, total score of Judgment of Line Orientation as a measure of visuospatial function, and total score of the Symbol Digit Modalities Test as a measure of processing speed.

Furthermore, we used the total score of University of Pennsylvania Smell Identification Test (UPSIT) as a measure of olfactory function and total score of the REM sleep Behavior Disorder Screening Questionnaire (RBDSQ) as a measure of dream-enacting behavior.

**Statistical analyses**

All statistical analyses were conducted using the statistical software R (version 4.0.5).^2,3^ R scripts used in this study was deposited in Mendeley data (For peer review, please refer to <https://data.mendeley.com/datasets/7s8sng9yn8/draft?a=93851b9a-c6ca-4bf7-88e5-dea68e5f37a4>).

We used Fisher's exact test, Wilcoxon rank sum test, and Pearson's Chi-squared test, as appropriate. Using the multivariate linear mixed-effects model with an interaction term, we adjusted for covariates and examined the interaction effect.^4^ As the covariates, we selected age, levodopa equivalent daily dose, disease duration, sex, and baseline scores of UPSIT and RBDSQ in reference to previous paper.^5^ In our model, each clinical parameter represented a response variable, whereas the predictor variables with fixed effects consisted of the duration of follow-up from the baseline, baseline lymphocyte count, the abovementioned covariates, and an interaction effect term between the first two predictor variables, and a predictor variable with random effects was each subject identification number. All continuous variables were Z-transformed in advance to aid in the interpretation of the results by putting different variables on the same scale. We primarily used the likelihood ratio test as a means to obtain the *P* value in a multivariate linear mixed-effects model.^6^ For computing the 95% CIs for each estimate, we used the profile CIs.^4^ Sensitivity analyses were conducted based on various follow-up periods and missing values which were corrected using the multiple imputation method.^7^

**Data availability**

All data used in this study are available in the PPMI database (<http://www.ppmi-info.org/data>). The R scripts used in this study is deposited in Mendeley Data and will be freely available upon publication (<https://doi.org/10.17632/7s8sng9yn8.1>; For peer review, please refer to <https://data.mendeley.com/datasets/7s8sng9yn8/draft?a=93851b9a-c6ca-4bf7-88e5-dea68e5f37a4>).

**Text S2.** Additional discussion regarding the importance of APOE ε4 allele in blood brain barrier dysfunction and circulating T cells in PD pathogenesis.

**The role of APOE ε4 allele in blood brain barrier (BBB) dysfunction**

Accumulating evidence showed that APOE ε4 allele drives BBB dysfunction.^8^ In mice, BBB dysfunction has been shown to cause neuronal degeneration by allowing toxic blood products to pass through the BBB and accumulate in neuronal cells.^9^ Consistently, in humans, BBB dysfunction has been shown to precede clinical symptoms and in Alzheimer’s disease and not related Aβ or tau pathology.^10^

**The role of circulating T cells in PD pathogenesis**

Accumulating evidence showed an enhancement in the infiltration of lymphocytes, especially T cells, into areas of the brain that are known to be vulnerable in PD.^11–14^ Importantly, an active role of T cells, especially circulating T cells, in the pathogenesis of PD is collectively suggested by observations that T cell infiltration even precedes abnormal α-synuclein deposition with infiltrated T cells being often observed in the vicinity of blood vessels, that T cells autoreactive to α-synuclein peptides are present in the blood vessels even from 10-years before the diagnosis, and that ablation of T cells markedly attenuates chemically-induced dopaminergic cell death in mice.^11,13–17^ Furthermore, T cell counts in the amygdala correlates both with α-synuclein and tau pathology in the same region and are associated with cognitive status in PD,^12^ indicating the critical role of T cells in the cortical and limbic pathology driving the cognitive impairment in PD.

**Text S3.** Additional discussion regarding limitations of our study.

There are several limitations to our study. First, our study did not adjust for comorbidities or the effect of drugs prescribed for comorbidities. This is because, given that not all diseases and drugs that affect lymphocyte counts have been identified, it is difficult to set inclusion criteria that, if inappropriate, could lead to selection bias. Second, the study was just observational. Third, our study excludes large number of patients included in the original PPMI study. However, this should not lead to a selection bias, as most of the excluded patients simply did not have information on their APOE genotype. Finally, our study lacked the information related to lymphocyte subtypes, which prohibited us from determining which cell type played the most critical role in our observations.

**SUPPLEMENTARY FIGURE**

**Supplementary Fig 1.** Study flowchart. Note that the majority of patients excluded from this study were due to lack of information on APOE genotype.
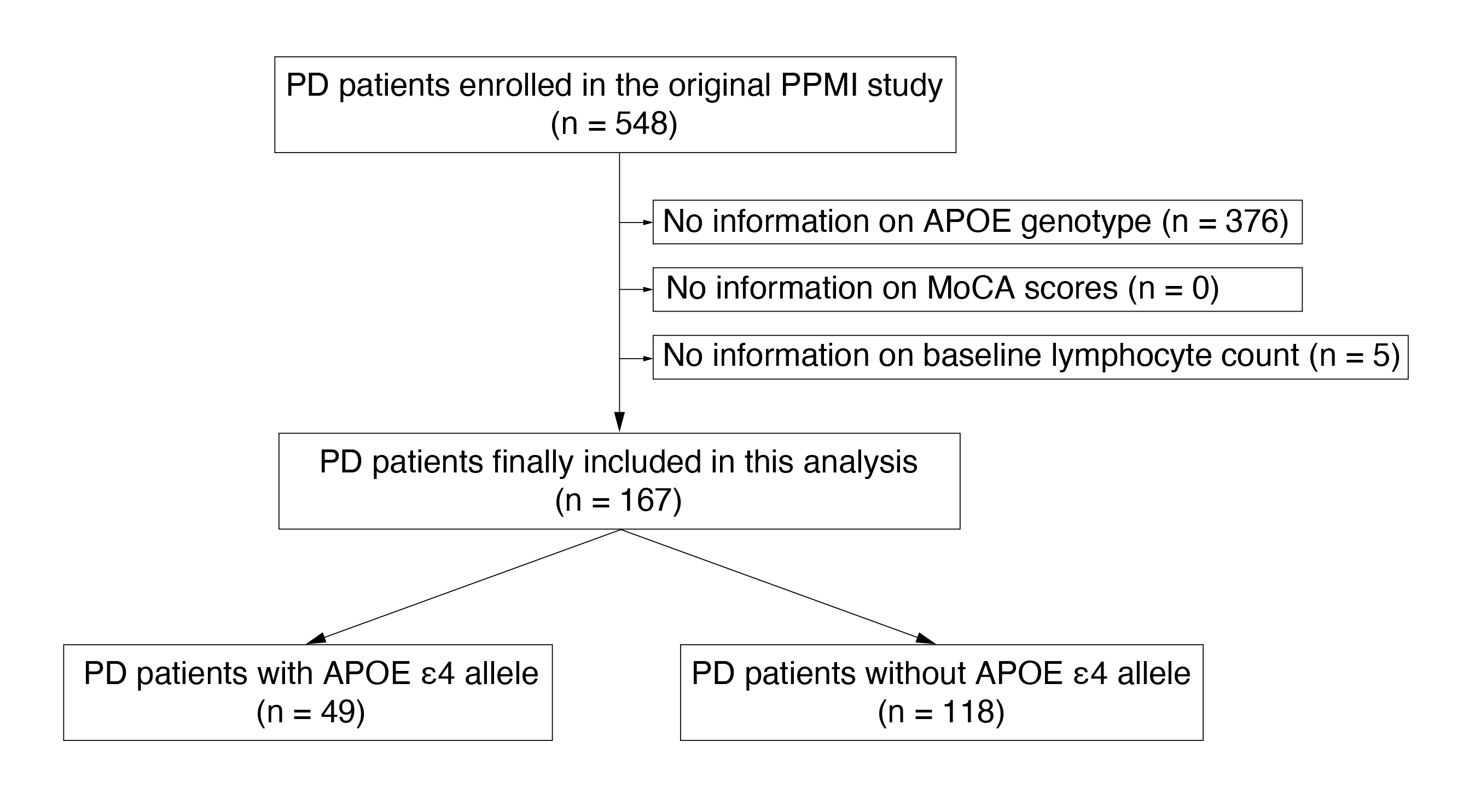


**SUPPLEMENTARY TABLES**

**SUPPLEMENTARY TABLES**

**Table S1.** Number of patients followed up for at least the described number of years. Since loss to follow-up of >20% poses serious threats to study validity, we primarily used 2-years as the follow-up period in this study.

| **Follow-up years** | **Number of PD patients** | **Percentage** |
| --- | --- | --- |
| 0 (Baseline) | 167 | 100 % |
| 1 | 145 | 86.8% |
| 2 | 136 | 81.4% |
| 3 | 121 | 72.5% |
| 4 | 117 | 70.1% |
| 5 | 106 | 63.5% |
| 6 | 92 | 55.1% |
| 7 | 83 | 49.7% |
| 8 | 75 | 44.9% |
| 9 | 12 | 7.2% |

**Table S2.** Characteristics of enrolled PD patients at the baseline and follow-ups.

|  | **At the baseline** | | **At 1 year follow up** | | **At 2 year follow up** | |
| --- | --- | --- | --- | --- | --- | --- |
|  | **APOE ε4 carrier**, N = 49 | **Non-carrier**,  N = 118 | **APOE ε4 carrier**, N = 39 | **Non-carrier**,  N = 94 | **APOE ε4 carrier**, N = 34 | **Non-carrier**,  N = 90 |
| ApoE Genotype |  |  |  |  |  |  |
| ε2/ε2 | – | 3.0 (2.5%) | – | 3.0 (3.2%) | – | 3.0 (3.3%) |
| ε2/ε3 | – | 22.0 (18.6%) | – | 18.0 (19.1%) | – | 17.0 (18.9%) |
| ε2/ε4 | 3.0 (6.1%) | – | 2.0 (5.1%) | – | 2.0 (5.9%) | – |
| ε3/ε3 | – | 93.0 (78.8%) | – | 73.0 (77.7%) | – | 70.0 (77.8%) |
| ε3/ε4 | 43.0 (87.8%) | – | 34.0 (87.2%) | – | 30.0 (88.2%) | – |
| ε4/ε4 | 3.0 (6.1%) | – | 3.0 (7.7%) | – | 2.0 (5.9%) | – |
| Age (years) | 59.0 (52.0, 65.0) | 63.0 (54.0, 68.0) | 60.0 (53.0, 66.0) | 63.5 (55.0, 69.0) | 61.0 (54.0, 67.0) | 65.0 (56.0, 71.0) |
| Sex, Male (%) | 35.0 (71.4%) | 75.0 (63.6%) | 27.0 (69.2%) | 61.0 (64.9%) | 24.0 (70.6%) | 60.0 (66.7%) |
| Disease duration from the diagnosis (years) | 0.0 (0.0, 1.0) | 0.0 (0.0, 1.0) | 2.0 (1.0, 3.0) | 2.0 (1.0, 2.0) | 3.0 (2.0, 4.0) | 3.0 (2.0, 3.0) |
| Hoen-Yahr stage | 2.0 (1.0, 2.0) | 2.0 (1.0, 2.0) | 2.0 (1.0, 2.0) | 2.0 (1.0, 2.0) | 2.0 (2.0, 2.0) | 2.0 (1.0, 2.0) |
| Missing | 6 | 9 | 4 | 5 | 4 | 14 |
| Baseline lymphocyte count (× 10^3^/μL) | 1.7 (1.4, 1.9) | 1.7 (1.4, 2.0) | 1.7 (1.4, 1.9) | 1.7 (1.4, 2.0) | 1.7 (1.4, 1.9) | 1.7 (1.4, 2.0) |
| MoCA Total score | 28.0 (26.0, 29.0) | 28.0 (27.0, 29.0) | 27.0 (25.0, 29.0) | 27.0 (25.0, 28.0) | 26.5 (23.0, 28.0) | 27.0 (25.0, 29.0) |
| HVLT-R delayed recall T score | 44.5 (39.0, 54.8) | 45.0 (37.8, 53.0) | 45.0 (37.5, 55.0) | 46.5 (37.2, 54.0) | 44.0 (36.2, 55.8) | 48.0 (38.5, 55.0) |
| Missing | 7 | 10 | 0 | 0 | 0 | 0 |
| JLO total score | 27.0 (24.0, 28.0) | 28.0 (24.0, 30.0) | 26.0 (23.0, 28.0) | 26.0 (24.0, 28.0) | 26.0 (24.0, 28.0) | 28.0 (24.0, 28.0) |
| Missing | 7 | 10 | 0 | 0 | 0 | 0 |
| SDMT total score | 40.5 (37.0, 45.8) | 44.0 (38.0, 48.0) | 41.0 (36.0, 49.5) | 42.0 (35.2, 48.0) | 41.5 (31.2, 48.0) | 41.5 (35.0, 47.0) |
| Missing | 7 | 10 | 0 | 0 | 0 | 0 |
| MDS-UPDRS part3 off score | 22.0 (15.5, 28.5) | 20.0 (15.0, 26.0) | 29.0 (18.5, 37.0) | 24.5 (16.8, 31.2) | 33.5 (22.2, 41.5) | 25.0 (18.0, 35.5) |
| Missing | 6 | 9 | 4 | 6 | 4 | 15 |
| UPSIT total score | 20.0 (14.0, 29.0) | 22.0 (17.0, 29.0) | – | – | – | – |
| Missing | 8 | 10 | 39 | 94 | 34 | 90 |
| RBDSQ total score | 6.0 (5.0, 9.0) | 5.0 (3.0, 7.0) | 6.0 (4.0, 8.0) | 5.0 (3.0, 7.0) | 7.0 (5.0, 9.0) | 5.5 (3.0, 8.0) |

Values are presented as median (interquartile range). Note that the number of patients evaluated at 1 and 2 years is different from the number of patients who were followed up for more than 1 and 2 years, respectively, because some patients skipped evaluations at some predefined time points.

Abberivations: ApoE, Apolipoprotein E; MoCA, Montreal Cognitive Assessment; HVLT-R, Hopkins Verbal Learning Test-Revised; JLO, Judgment of Line Orientation; SDMT, Symbol Digit Modalities Test; MDS-UPDRS, Movement Disorders Society-sponsored revision of the Unified Parkinson's disease rating scale.

**Table S3** Baseline characteristics of two groups of PD patients dichotomized by median baseline lymphocyte count.

|  | **APOE ε4 carrier** | |  | **APOE ε4 non-carrier** | |  |
| --- | --- | --- | --- | --- | --- | --- |
|  | **Lower**, N = 25 | **Higher**, N = 24 | ***P* value** | **Lower**, N = 58 | **Higher**, N = 60 | ***P* value** |
| Baseline lymphocyte count (× 10^3^/μL) | 1.4 (1.2, 1.4) | 1.9 (1.8, 2.2) | <0.001 | 1.4 (1.2, 1.6) | 2.0 (1.8, 2.3) | <0.001 |
| ApoE Genotype |  |  | >0.99 |  |  | 0.86 |
| ε2/ε2 | – | – |  | 1.0 (1.7%) | 2.0 (3.3%) |  |
| ε2/ε3 | – | – |  | 10.0 (17.2%) | 12.0 (20.0%) |  |
| ε2/ε4 | 1.0 (4.0%) | 2.0 (8.3%) |  | – | – |  |
| ε3/ε3 | – | – |  | 47.0 (81.0%) | 46.0 (76.7%) |  |
| ε3/ε4 | 22.0 (88.0%) | 21.0 (87.5%) |  | – | – |  |
| ε4/ε4 | 2.0 (8.0%) | 1.0 (4.2%) |  | – | – |  |
| Age (years) | 62.0 (53.0, 68.0) | 56.5 (49.0, 64.2) |  | 63.0 (56.2, 67.8) | 62.0 (52.8, 69.0) | 0.72 |
| Sex, Male (%) | 19.0 (76.0%) | 16.0 (66.7%) | 0.47 | 42.0 (72.4%) | 33.0 (55.0%) | 0.049 |
| Disease duration from the diagnosis (years) | 0.0 (0.0, 1.0) | 0.0 (0.0, 1.0) |  | 0.0 (0.0, 1.0) | 0.5 (0.0, 1.0) | 0.37 |
| Hoen-Yahr stage | 2.0 (1.0, 2.0) | 2.0 (1.0, 2.0) |  | 2.0 (1.0, 2.0) | 2.0 (1.0, 2.0) | 0.37 |
| Missing | 5 | 1 |  | 1 | 8 |  |
| Levodopa equivalent daily dose (mg) | 0.0 (0.0, 0.0) | 0.0 (0.0, 0.0) | >0.99 | 0.0 (0.0, 0.0) | 0.0 (0.0, 0.0) | >0.99 |
| MoCA Total score | 28.0 (26.0, 28.0) | 27.5 (26.8, 29.2) | 0.55 | 28.0 (27.0, 29.0) | 28.0 (27.0, 29.0) | 0.90 |
| HVLT-R delayed recall T score | 44.5 (36.8, 55.2) | 44.5 (40.0, 54.0) | 0.81 | 47.0 (39.0, 53.0) | 45.0 (36.5, 52.0) | 0.41 |
| Missing | 5 | 2 |  | 1 | 9 |  |
| JLO total score | 26.0 (21.5, 28.0) | 28.0 (26.0, 28.0) | 0.27 | 28.0 (24.0, 30.0) | 28.0 (24.0, 30.0) | 0.45 |
| Missing | 5 | 2 |  | 1 | 9 |  |
| SDMT total score | 40.0 (34.8, 43.2) | 42.5 (37.2, 49.5) | 0.13 | 43.0 (38.0, 48.0) | 44.0 (38.0, 48.0) | 0.98 |
| Missing | 5 | 2 |  | 1 | 9 |  |
| MDS-UPDRS part3 off score | 22.0 (16.8, 35.2) | 21.0 (15.0, 27.0) | 0.41 | 19.0 (14.0, 26.0) | 21.0 (16.0, 25.2) | 0.41 |
| Missing | 5 | 1 |  | 1 | 8 |  |
| UPSIT total score | 16.0 (12.5, 25.5) | 23.5 (16.2, 33.0) | 0.06 | 22.0 (17.0, 28.0) | 24.0 (17.0, 29.0) | 0.58 |
| Missing | 5 | 3 |  | 1 | 9 |  |
| RBDSQ total score | 6.0 (4.8, 9.0) | 6.0 (5.0, 9.0) | 0.80 | 4.0 (3.0, 6.0) | 6.0 (3.0, 7.0) | 0.16 |
| Missing | 4 | 2 |  | 1 | 9 |  |

Values are presented as median (interquartile range). *P* value was obtained by Fisher's exact test, Wilcoxon rank sum test, and Pearson's Chi-squared test, as appropriate. Note that the number of patients evaluated at 1 and 2 years is different from the number of patients who were followed up for more than 1 and 2 years, respectively, because some patients skipped evaluations at some predefined time points.

Abberivations: ApoE, Apolipoprotein E; MoCA, Montreal Cognitive Assessment; HVLT-R, Hopkins Verbal Learning Test-Revised; JLO, Judgment of Line Orientation; SDMT, Symbol Digit Modalities Test; MDS-UPDRS, Movement Disorders Society-sponsored revision of the Unified Parkinson's disease rating scale; UPSIT, University of Pennsylvania Smell Identification Test; RBDSQ, Rapid-eye-movement sleep Behavior Disorder Screening Questionnaire.

**Table S4.** Sensitivity analyses with various follow-up periods.

| **Follow-up years** | **Characteristics** | **Estimates** | **95%CI, low** | **95%CI, high** |
| --- | --- | --- | --- | --- |
| 3 | All PD patients | 0.04 | -0.02 | 0.10 |
|  | ApoE ε4 carrier | 0.14 | 0.03 | 0.25 |
|  | ApoE ε4 non-carrier | -0.00 | -0.08 | 0.07 |
| 4 | All PD patients | 0.02 | -0.03 | 0.08 |
|  | ApoE ε4 carrier | 0.11 | 0.00 | 0.21 |
|  | ApoE ε4 non-carrier | -0.02 | -0.09 | 0.05 |
| 5 | All PD patients | 0.01 | -0.05 | 0.06 |
|  | ApoE ε4 carrier | 0.09 | -0.00 | 0.19 |
|  | ApoE ε4 non-carrier | -0.03 | -0.09 | 0.03 |
| 6 | All PD patients | 0.02 | -0.03 | 0.07 |
|  | ApoE ε4 carrier | 0.09 | 0.00 | 0.18 |
|  | ApoE ε4 non-carrier | -0.01 | -0.07 | 0.05 |
| 7 | All PD patients | 0.02 | -0.03 | 0.07 |
|  | ApoE ε4 carrier | 0.11 | 0.02 | 0.20 |
|  | ApoE ε4 non-carrier | -0.02 | -0.08 | 0.03 |
| 8 | All PD patients | 0.01 | -0.04 | 0.05 |
|  | ApoE ε4 carrier | 0.11 | 0.02 | 0.20 |
|  | ApoE ε4 non-carrier | -0.04 | -0.09 | 0.02 |
| 9 | All PD patients | 0.01 | -0.04 | 0.05 |
|  | ApoE ε4 carrier | 0.11 | 0.02 | 0.20 |
|  | ApoE ε4 non-carrier | -0.03 | -0.09 | 0.02 |

**Table S5.** Summary values obtained from sensitivity analyses using the multiple imputation for missing data with expectation-maximization with bootstrapping algorithm (100 times).

|  | **Median** | **2.5 %** | **97.5 %** |
| --- | --- | --- | --- |
| **Estimate** | 0.172 | 0.169 | 0.174 |
| **2.5 %** | 0.045 | 0.0476 | 0.0495 |
| **97.5 %** | 0.294 | 0.297 | 0.298 |

SUPPLEMENTARY REFERENCES

1. Dettori JR. Loss to follow-up. *Evid-Based Spine-Care J*. 2011; 2: 7–10. doi:10.1055/s-0030-1267080

2. Tsukita K, Tachibana N, Hamano T. Appropriate assessment method of 123I-MIBG myocardial scintigraphy for the diagnosis of Lewy body diseases and idiopathic REM sleep behavior disorder. *J Neurol*. 2020; 267: 3248–3257. doi:10.1007/s00415-020-09992-0

3. Tsukita K, Taguchi T, Sakamaki-Tsukita H, Tanaka K, Suenaga T. The vagus nerve becomes smaller in patients with Parkinson’s disease: A preliminary cross-sectional study using ultrasonography. *Parkinsonism Relat Disord*. 2018; 55: 148–149. doi:10.1016/j.parkreldis.2018.06.002

4. Bates D, Mächler M, Bolker B, Walker S. Fitting Linear Mixed-Effects Models Using lme4. *J Stat Softw*. 2015; 67. doi:10.18637/jss.v067.i01

5. Schrag A, Siddiqui UF, Anastasiou Z, Weintraub D, Schott JM. Clinical variables and biomarkers in prediction of cognitive impairment in patients with newly diagnosed Parkinson’s disease: a cohort study. *Lancet Neurol*. 2017; 16: 66–75. doi:10.1016/S1474-4422(16)30328-3

6. Luke SG. Evaluating significance in linear mixed-effects models in R. *Behav Res Methods*. 2017; 49: 1494–1502. doi:10.3758/s13428-016-0809-y

7. Honaker J, King G, Blackwell M. Amelia II: A Program for Missing Data. *J Stat Softw*. 2011; 45. doi:10.18637/jss.v045.i07

8. Montagne A, Zhao Z, Zlokovic BV. Alzheimer’s disease: A matter of blood-brain barrier dysfunction? *J Exp Med*. 2017; 214: 3151–3169. doi:10.1084/jem.20171406

9. Bell RD, Winkler EA, Singh I, et al. Apolipoprotein E controls cerebrovascular integrity via cyclophilin A. *Nature*. 2012; 485: 512–516. doi:10.1038/nature11087

10. Montagne A, Nation DA, Sagare AP, et al. APOE4 leads to blood-brain barrier dysfunction predicting cognitive decline. *Nature*. 2020; 581: 71–76. doi:10.1038/s41586-020-2247-3

11. Galiano-Landeira J, Torra A, Vila M, Bové J. CD8 T cell nigral infiltration precedes synucleinopathy in early stages of Parkinson’s disease. *Brain*. 2020; 143: 3717–3733. doi:10.1093/brain/awaa269

12. Kouli A, Camacho M, Allinson K, Williams-Gray CH. Neuroinflammation and protein pathology in Parkinson’s disease dementia. *Acta Neuropathol Commun*. 2020; 8: 211. doi:10.1186/s40478-020-01083-5

13. Brochard V, Combadière B, Prigent A, et al. Infiltration of CD4+ lymphocytes into the brain contributes to neurodegeneration in a mouse model of Parkinson disease. *J Clin Invest*. 2009; 119: 182–192. doi:10.1172/JCI36470

14. Sommer A, Marxreiter F, Krach F, et al. Th17 Lymphocytes Induce Neuronal Cell Death in a Human iPSC-Based Model of Parkinson’s Disease. *Cell Stem Cell*. 2018; 23: 123–131.e6. doi:10.1016/j.stem.2018.06.015

15. Sulzer D, Alcalay RN, Garretti F, et al. T cells from patients with Parkinson’s disease recognize α-synuclein peptides. *Nature*. 2017; 546: 656–661. doi:10.1038/nature22815

16. Lindestam Arlehamn CS, Dhanwani R, Pham J, et al. α-Synuclein-specific T cell reactivity is associated with preclinical and early Parkinson’s disease. *Nat Commun*. 2020; 11: 1875. doi:10.1038/s41467-020-15626-w

17. Tan JSY, Chao YX, Rötzschke O, Tan E-K. New Insights into Immune-Mediated Mechanisms in Parkinson’s Disease. *Int J Mol Sci*. 2020; 21: E9302. doi:10.3390/ijms21239302
